# The Efficacy of Probiotics, Prebiotics, and Synbiotics in Patients Who Have Undergone Abdominal Operation, in Terms of Bowel Function Post-Operatively: A Network Meta-Analysis

**DOI:** 10.3390/jcm12124150

**Published:** 2023-06-20

**Authors:** Orestis Ioannidis, Christos Chatzakis, Maria Tirta, Elissavet Anestiadou, Konstantinos Zapsalis, Savvas Symeonidis, Stefanos Bitsianis, Efstathios Kotidis, Manousos George Pramateftakis, Ioannis Mantzoros, Stamatios Angelopoulos

**Affiliations:** 1 Fourth Surgical Department, Aristotle University of Thessaloniki, 54124 Thessaloniki, Greeceelissavetxatz@gmail.com (E.A.); sbitsiani@gmail.com (S.B.); saggelopoulos@auth.gr (S.A.); 2 Second Department of Obstetrics and Gynecology, Aristotle University of Thessaloniki, 54124 Thessaloniki, Greece; 3 Department of Hygiene, Epidemiology and Medical Statistics, National and Kapodistrian University of Athens, 11527 Athens, Greece

**Keywords:** probiotics, prebiotics, synbiotics, bowel function, abdominal operation

## Abstract

Background: Abdominal operations may lead to post-operative bowel dysfunction, while administration of probiotics, prebiotics and synbiotics may limit its manifestation. Τhe study aimed to assess the efficacy of probiotics, prebiotics and synbiotics in patients who undergone abdominal operation, in terms of bowel function post-operatively. Methods: PubMed, Scopus, Cochrane Central Register of Controlled Trials (Central), Embase, US Registry of clinical trials, and sources of grey literature were searched. The relative effect sizes were estimated, and we obtained the relative ranking of the interventions using cumulative ranking curves. Results: In total, 30 studies were included in the analysis. For the outcome of post-operative ileus, probiotics was superior to placebo/no intervention (relative risk, RR: 0.38; 95%CI: 0.14–0.98) with the highest SUCRA (surface under the cumulative ranking) value (92.1%). For time to first flatus, probiotics (MD: −0.47; 95%CI: −0.78 to −0.17) and synbiotics (MD: −0.53; 95%CI: −0.96 to −0.09) were superior to placebo/no intervention. For time to first defecation and for post-operative abdominal distension probiotics were superior to placebo/no intervention. For post-operative hospitalization days, synbiotics were superior to placebo/no intervention (MD: −3.07; 95%CI: −4.80 to −1.34). Conclusions: Administration of probiotics in patients who had undergone abdominal surgery reduced the prevalence of post-operative ileus, time to first flatus, time to first defecation, and prevalence of post-operative abdominal distension. Synbiotics reduce time to first flatus and post-operative hospitalization days.

## 1. Introduction

Abdominal operations are commonly performed surgical procedures aimed at resolving various medical conditions. While these interventions are often successful in treating the underlying issues, they can also disrupt the natural balance of the intestinal microbiota, leading to post-operative complications such as impaired bowel function, intra-abdominal infections, and sepsis [1,2]. The importance of optimal post-operative recovery cannot be overstated, as it directly impacts patients’ quality of life, hospital stay length, and healthcare costs [3,4].

Probiotics, prebiotics, and synbiotics have emerged as potential therapeutic strategies to restore and enhance the intestinal microbiota, offering promising solutions for improving post-operative bowel function [5]. Adequate administration of live microorganisms called probiotics results in positive effects on health. [6]. They can include strains of Lactobacillus, Bifidobacterium, and other beneficial bacteria, which exert their effects by modulating the gut microbiota composition and promoting a healthy intestinal environment [7,8]. In contrast, prebiotics are non-digestible compounds that selectively promote the growth and function of beneficial bacteria in the intestinal tract [9]. Combining probiotics and prebiotics, synbiotics adopt a dual approach, introducing beneficial bacteria while also providing the necessary nourishment for their growth [10].

The use of probiotics, prebiotics, and synbiotics has gained significant attention in various gastrointestinal conditions [11]. Probiotics exert their effects through various mechanisms, such as stimulating the production of anti-inflammatory cytokines and producing bacteriocins that hinder the attachment of harmful bacteria to the epithelial lining and the production of virulence factors. They also help prevent the movement of bacteria across tight junctions [12]. According to a recent meta-analysis, probiotics and synbiotics have been found to decrease the likelihood of infection complications following abdominal surgery [13]. Furthermore, probiotics and synbiotics may enhance gastrointestinal motility [14]. However, their specific role in enhancing post-operative bowel function remains underexplored. Understanding the efficacy of these interventions in the context of abdominal surgery is crucial for optimizing patient outcomes and advancing surgical care.

This network meta-analysis aims to evaluate the effectiveness of probiotics, prebiotics, and synbiotics in patients who have undergone abdominal operations, specifically focusing on post-operative bowel function.

## 2. Materials and Methods

### 2.1. Reporting Guideline and Registration

Conducting this meta-analysis adhered to the guidelines outlined in the PRISMA extension statement for network meta-analyses [15], and the study has been registered with PROSPERO (CRD42020160433).

### 2.2. Inclusion and Exclusion Criteria

Randomized controlled trials (RCTs) comparing the effect of probiotics, prebiotics, and synbiotics vs. placebo or no intervention on the bowel function after an abdominal operation in adult patients (>18 years old) were eligible for inclusion. Abdominal operation definition included surgeries that involve any gastrointestinal organ. No language restrictions were imposed, and studies were not excluded based on their publication date. However, studies that involved patients who did not undergo an abdominal operation were excluded.

### 2.3. Primary and Secondary Outcome Measures

The primary outcomes of the study were the post-operative ileus and the time to first flatus in patients who had undergone a gastrointestinal operation and received probiotics, prebiotics, prebiotics, synbiotic, or placebo/no intervention. The secondary outcomes of the study were the time to first defecation, the length of post-operative hospital stay, and the abdominal distension in patients who had undergone a gastrointestinal operation and received probiotics, prebiotics, synbiotic, or placebo/no intervention.

### 2.4. Search Methods

The identification of eligible studies was carried out using a pre-defined search strategy in electronic databases published from inception to 17 March 2023 [PubMed, Embase, Scopus, US Registry of clinical trials (www.clinicaltrials.gov)], Cochrane Central Register of Controlled Trials (Central), and sources of grey literature, using combinations of the terms “probiotics”, “prebiotics”, “synbiotics”, “surgery”, “operation”, “gastrointestinal”, “colorectal”,” abdominal”, and “pancreatic” (Table S1). In addition to the main search, the references of the retrieved articles were reviewed, and an automated search was conducted using PubMed’s “search for related articles” feature to supplement the searches. All studies were carefully compared to prevent the inclusion of duplicate or overlapping samples. In cases where overlap occurred, the study with the highest number of cases was included.

### 2.5. Study Selection

Two reviewers (CC and MT) independently assessed the eligibility of all the identified studies based on the aforementioned criteria. In cases of disagreements between the reviewers, a third reviewer (OI) was consulted for resolution through arbitration.

### 2.6. Data Extraction

The following information was extracted during data extraction from each study: first author, year, sample size, age, gender, primary disease, type of surgery, study type, duration of treatment, intervention details, data from the control group, and outcomes. Two reviewers (OI and CC) independently assessed the quality of each study. The study characteristics of each included study were evaluated using a predefined data extraction form. In instances of disagreement, consensus was reached through discussions between the two reviewers until an agreement was reached.

### 2.7. Risk of Bias

The included studies were objectively assessed for their risk of bias in terms of internal validity using the Cochrane “risk of bias” tool 2 [16]. The factors that were evaluated were random sequence generation, allocation concealment, blinding of participants, personnel and assessors, incomplete outcome data, and selective reporting. Five distinct domains were examined to identify biases related to randomization, deviations from intended interventions, missing outcome data, outcome measurements, and the selection of reported results. Studies were categorized as “low risk” when all the items were rated as having a low risk of bias across all domains. Studies were categorized as having “some concerns” if one item in a domain was rated as having some concerns. Studies were categorized as “high risk” if at least one item in a domain was rated as high risk or multiple items in domains were rated as having some concerns.

### 2.8. Geometry of the Networks

For each outcome (post-operative ileus, time to first flatus, time to first defecation, length of post-operative hospital stay, and abdominal distension), a network plot was created to include all groups that received probiotics, prebiotics, synbiotics, or placebo/no intervention. Each group was represented by nodes, and the comparisons between them were represented by edges. The size of a node was proportionate to the number of patients, while the thickness of the edges reflected the number of studies evaluating each intervention (probiotics, prebiotics, synbiotics, or placebo/no intervention). The network plots were generated using the “netgraph” command from the “netmeta” package [17] in R (R: a language environment for statistical computing, R Foundation for Statistical Computing, Vienna, Austria).

### 2.9. Assessment of Transitivity

Transitivity, which is a crucial assumption in network meta-analysis, suggests that valid comparisons between two interventions can be made through connected indirect routes that involve one or more intermediate comparators. Transitivity can be assessed by comparing the distribution of potential effect modifiers among the direct comparisons available in the network [18]. Information regarding patient and study characteristics that may act as effect modifiers were collected and are presented in Table S3.

### 2.10. Statistical Analysis

Direct estimates were obtained using a comparison-specific random-effects model. Subsequently, a random-effects network meta-analysis was conducted to simultaneously compare the relative effectiveness of all interventions [19]. A common heterogeneity (τ) was assumed across all comparisons and compared with previously derived empirical distributions for heterogeneity [20]. Heterogeneity was assessed using Cochran’s Q test and the I-squared (I²) statistic. Heterogeneity was categorized as low, moderate, or substantial when the value of I² was less than 25%, 50%, or greater than or equal to 75%, respectively. In cases where heterogeneity was detected (I² ≥ 50 or with a clearly identified reason), a sensitivity analysis excluding the relevant trials was performed. For all possible pairwise comparisons, the mean difference (MD) with a 95% confidence interval (CI) was estimated using a multivariate meta-analysis approach, which treats different comparisons in studies as separate outcomes and accounts for correlation introduced by multi-arm trials [16]. The network meta-analysis models were conducted using the netmeta package in R. The analyses were performed on an intention-to-treat basis. To assess the ranking probabilities of each intervention, cumulative ranking curves were plotted, and the surface under these curves (surface under the cumulative ranking, SUCRA) was calculated. SUCRA is expressed as a percentage and represents the effectiveness of an intervention compared to a theoretical intervention that is always assumed to be the best without uncertainty. A higher SUCRA value indicates a better rank for the intervention [21]. Contribution plots were created to evaluate the influence of each direct comparison on the network estimates and the overall network.

### 2.11. Assessment of Inconsistency

To assess the consistency of intervention effects (i.e., the agreement between direct and indirect evidence), an inconsistency plot was generated using the “netheat” command in R. In each loop, the inconsistency factor was calculated as the ratio of the two mean differences obtained from the direct and indirect evidence for a specific comparison within the loop. Values close to 1 indicate statistical agreement between the two sources of evidence. If the 95% confidence interval does not include unity (1), significant inconsistency in a loop is detected. This analysis was performed assuming a common heterogeneity parameter across all loops in the network, as estimated from the network meta-analysis model.

### 2.12. Assessment of Small-Study Effects

To account for the potential influence of small studies and mitigate publication bias, the effect was evaluated using a comparison-adjusted funnel plot. This plot considered the estimated effects of studies for different comparisons within the network.

## 3. Results

### 3.1. Search Results

**Figure 1:**
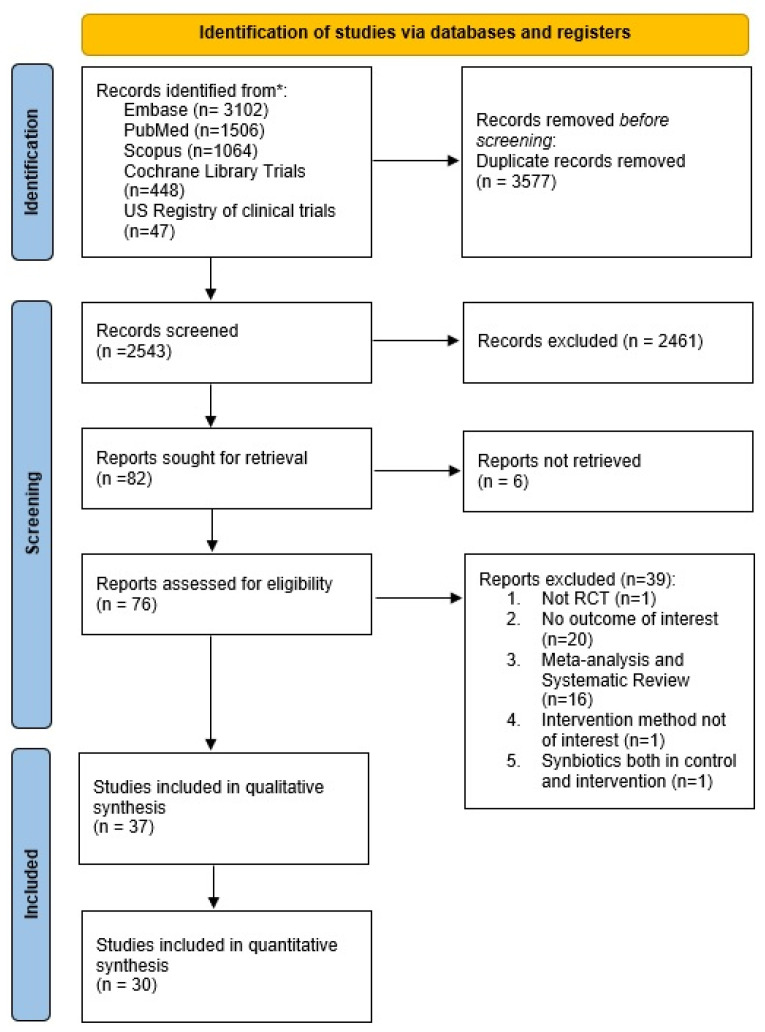
Flowchart of the selection procedure. * Last search 17 March 2023.

The initial electronic search yielded a total of 6167 records. The selection process is illustrated in Figure 1. After excluding 39 studies with their respective reasons, a total of 37 studies published between 2002 and 2021 were included in the qualitative synthesis. The characteristics of these included studies can be found in Table S3. Out of these, 30 studies [22,23,24,25,26,27,28,29,30,31,32,33,34,35,36,37,38,39,40,41,42,43,44,45,46,47,48,49,50,51,52,53,54,55,56,57,58] were included in the quantitative synthesis. Details on the 39 excluded studies and the reasons for their exclusion are provided in Table S2. Seven studies were included in the systematic review but not in the quantitative synthesis. Among them, four studies did not provide the necessary descriptive statistic measures [31,36,40,45], and in the remaining three studies, the outcome measures did not match the predefined primary and secondary outcomes [27,46,53]. Ultimately, the quantitative synthesis (meta-analysis) consisted of 30 studies involving a total of 2267 patients.

### 3.2. Geometry of the Networks

**Figure 2:**
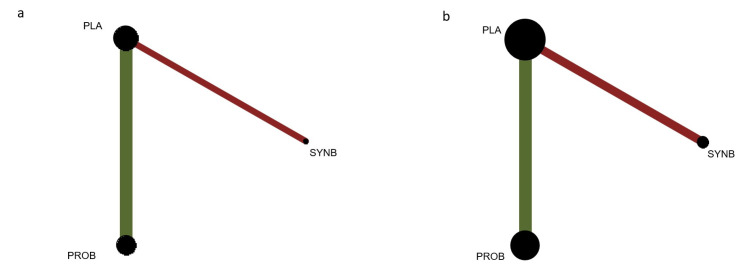
(**a**) Network plot for post-operative ileus and (**b**) network plot for the time to first flatus. Comparison of probiotic supplementation versus placebo/no intervention (green) and comparison of synbiotics versus placebo/no intervention (red).

Figure 2a displays the network plot for post-operative ileus. In this analysis, all interventions were pairwise tested in five studies. The most frequently examined comparison involved probiotic supplementation versus placebo/no intervention, which was evaluated in four studies [28,33,49,52]. Among these studies, the largest number of patients were included in the probiotic supplementation versus placebo comparison, involving 217 patients across four studies [28,33,49,52]. Placebo/no intervention was administered to 303 patients in a total of five studies [28,33,41,49,52].

Figure 2b illustrates the network plot for the time to first flatus. In this analysis, all interventions were pairwise tested in ten studies. The most common comparison was probiotic supplementation versus placebo/no intervention, which was investigated in seven studies [44,48,51,55,56,58,59]. Among these studies, the largest number of patients were included in the probiotic supplementation versus placebo comparison, involving 521 patients across seven studies [44,48,51,55,56,58,59]. Placebo/no intervention was administered to 350 patients across ten studies [32,44,47,48,51,55,56,57,58,59].

### 3.3. Risk of Bias

The risk of bias is presented in Table S4. Twenty-five of the studies conducted an appropriate randomization process [22,23,26,27,28,29,30,31,34,36,37,38,39,40,41,43,44,45,46,50,52,53,54,55,57]. In 18 studies [22,23,25,26,27,29,31,34,36,38,39,43,46,50,52,54,55,58], deviations from intended interventions were evaluated and deemed to pose a low risk of bias. Regarding missing outcome data, outcome measurement, and selection of the reported result, the risk of bias was assessed as low in all studies, except for seven studies [22,23,31,34,36,38,46]. The overall risk of nine studies was assessed as a low risk of bias [26,27,29,39,43,50,52,54,55], 16 studies with a high risk of bias [23,24,31,32,33,34,35,42,45,47,48,49,51,53,56,58], and 12 with some concerns [23,25,28,30,36,37,38,40,41,44,46,57].

### 3.4. Assessment of Transitivity and Inconsistency

Among studies that compared more than one intervention (Table S3), no discrepancies were found in terms of study and participant characteristics, as well as the definition of intervention and outcomes. In order to assess transitivity, inconsistency was evaluated, but no evidence of inconsistency was observed.

### 3.5. Primary Outcomes

For post-operative ileus, the administration of probiotics was found to be superior to placebo/no intervention (relative risk, RR: 0.38; 95% CI: 0.14–0.98) (Table 1). In terms of relative ranking, probiotics achieved the highest SUCRA value of 92.1%, followed by synbiotics with 40.2%. Placebo/no intervention was found to be the least effective option (Table 2).

In relation to the time to first flatus, both probiotics (MD: −0.47; 95% CI: −0.78 to −0.17) and synbiotics (MD: −0.53; 95% CI −0.96 to −0.09) were superior to placebo/no intervention (Table 1). Regarding relative ranking, synbiotics had the highest SUCRA value (78.3%), followed by probiotics (71.2%), while placebo/no intervention was the least effective (Table 2).

### 3.6. Secondary Outcomes

For the outcome of time to first defecation, a total of nine studies contributed [17,22,30,41,42,43,44,45,53]. The most common comparison was probiotic supplementation versus placebo/no intervention, which was studied in seven studies. The largest number of patients were included in the probiotic supplementation versus placebo comparison, with a total of 558 patients across seven studies. Placebo/no intervention was given to 328 patients across nine studies. Probiotics demonstrated superiority over placebo/no intervention (MD: −0.70; 95% CI: −1.23 to −0.18) (Table 1). In terms of relative ranking, among the interventions, probiotics had the highest SUCRA value (91.1%), followed by synbiotics (39.5%), while placebo/no intervention was found to be the least effective (Table 2).

Six studies contributed to the outcome of post-operative abdominal distension [26,29,37,44,47,58]. The most common comparison was probiotic supplementation vs. placebo/no intervention (four studies). The most studied patients were included in probiotic supplementation vs. placebo (364 patients, four studies) and Placebo / no intervention was given in 223 patients (five studies). Probiotics were superior to placebo/no intervention (RR: 0.63; 95% CI: 0.48–0.82) (Table 1). Regarding relative ranking among interventions, synbiotics had the highest SUCRA value (79.4%), followed by probiotics (54.6%) and prebiotics (54.0%), while placebo/no intervention was the least effective (Table 2).

A total of 19 studies contributed to the outcome of post-operative hospitalization days [22,23,24,25,26,29,30,34,35,37,38,39,42,43,44,47,48,50,59]. The most common comparison in these studies was synbiotics supplementation versus placebo/no intervention, which was examined in 10 studies. The largest number of patients were included in the synbiotics supplementation versus placebo comparison, with a total of 584 patients across 10 studies. Placebo/no intervention was given to 583 patients across 17 studies. Synbiotics were found to be superior to placebo/no intervention (MD: −3.07; 95% CI: −4.80 to −1.34), as presented in Table 1. Regarding relative ranking among the interventions, synbiotics had the highest SUCRA value (91.6%), followed by prebiotics (54.7%) and probiotics (39.6%), while placebo/no intervention was the least effective (Table 2).

### 3.7. Small-Study Effects

The comparison-adjusted funnel plots demonstrated symmetric distribution for all outcomes, indicating the absence of a significant small study effect (Figure S1).

### 3.8. Quality of the Evidence

The overall quality of evidence, evaluated using GRADE criteria adapted for network meta-analysis, ranged from low to moderate. A detailed presentation of the evidence quality can be found in Table S5.

Study limitations: In all network estimates, the evidence was downgraded in the following cases: (a) by one level if more than 50% of the information originated from studies with “some concerns” or “high risk” of bias, and (b) by two levels if more than 50% of the information originated from studies with “high risk” of bias.

Indirectness: There were no differences observed in the baseline characteristics of the patients among the studies, hence no downgrading occurred.

Inconsistency: As there were no closed loops available, the point estimates of direct and indirect comparisons were assessed. The evidence was downgraded by one level if the prediction intervals extended across the line of no effect.

Imprecision: The evidence was downgraded by one level if the prediction intervals extended across the line of no effect.

Publication bias: The comparison-adjusted funnel plot exhibited symmetry for all outcomes (Figure S1). Therefore, no downgrading was necessary.

## 4. Discussion

In this study we showed that administration of probiotics in patients who had undergone abdominal surgery reduced the prevalence of post-operative ileus, the time to first flatus, the time to first defecation, and the prevalence of post-operative abdominal distension. Administration of synbiotics reduced both the time to first flatus and the duration of post-operative hospitalization. In terms of treatment’s ranking, none of the interventions were universally superior to the others (Table 2).

Post-operative recovery following abdominal operations can be a challenging and lengthy process [60,61]. However, recent advancements in the field of gut health have shed light on the potential benefits of utilizing prebiotics, probiotics, and their combination to decrease the time required for the return of important gastrointestinal functions, such as the passage of first flatulence and bowel movements [5].

Probiotics refer to live microorganisms that, when administered in sufficient quantities, provide a beneficial effect to the host [62]. These beneficial bacteria play a pivotal role in post-operative recovery by modulating gut microbiota, regulating immune responses, and enhancing intestinal barrier function [59,63,64]. Probiotics have been found to decrease the incidence of post-operative complications, including infection and inflammation, thus promoting faster recovery [65,66]. By colonizing the gastrointestinal tract with beneficial microorganisms, probiotics help in the efficient digestion and absorption of nutrients, facilitating early recovery of bowel function [67].

Prebiotics are non-digestible dietary fibers that selectively stimulate the growth and activity of beneficial bacteria in the gut [68]. They serve as a nourishing source for probiotic strains, aiding in the restoration of disrupted gut microbiota balance caused by surgical procedures [69]. By enhancing the growth of beneficial bacteria, prebiotics create a favorable environment for the re-establishment of gut homeostasis. This accelerated restoration of gut microbiota balance contributes to reduced post-operative recovery time.

The combination of prebiotics and probiotics exhibits a synergistic effect that surpasses the benefits achieved by each component alone. Prebiotics provide nourishment and a supportive environment for probiotics, enhancing their colonization and activity in the gut. In return, probiotics metabolize prebiotics into beneficial metabolites, such as short-chain fatty acids, which further promote intestinal health and enhance gut motility. This symbiotic relationship between prebiotics and probiotics accelerates the restoration of gut microbiota diversity, fostering a quicker return to normal bowel function after surgery [10,70].

In relation to post-operative ileus, its exact mechanism remains unclear and is likely influenced by a combination of various factors [71]. However, one prominent factor contributing to the inhibition of gastrointestinal motility is the overstimulation of the sympathetic nerve during surgery [72]. By supplementing with probiotics or synbiotics before and after the operation, it is possible to alleviate post-operative ileus by reducing the levels of pro-inflammatory molecules such as tumor necrosis factor-α, interleukin-6, C-reactive protein, and nitric oxide [73]. These inflammatory markers can contribute to the aggravation of post-operative ileus, and their modulation through the restoration of intestinal flora balance can be beneficial [73].

Furthermore, specific probiotic species such as Lactobacillus and Bifidobacterium have the ability to produce certain substances known as short-chain fatty acids (SCFAs) within the gut [74]. These SCFAs, including butyrate, acetate, and propionate, are generated through the fermentation process of dietary fiber by probiotic bacteria [75]. SCFAs serve as an essential energy source for the cells that line the colon and play a vital role in maintaining the overall health of the gut. They provide nourishment to the intestinal epithelial cells, promote their growth and differentiation, and contribute to the integrity of the intestinal barrier function. Additionally, SCFAs possess anti-inflammatory properties and help modulate immune responses, thereby benefiting the overall health of the gut [76]. Furthermore, SCFAs can influence the composition of the gut microbiota by selectively promoting the growth of beneficial bacteria while inhibiting the growth of harmful pathogens [76]. The metabolites produced by specific probiotic species have diverse effects on various aspects of gut function, including cellular energy metabolism, maintenance of the gut barrier, immune modulation, and modulation of the composition of the gut microbiota [77,78].

This study represents the first network meta-analysis conducted to evaluate the effects of probiotics, prebiotics, and synbiotics on post-operative bowel function in patients who have undergone abdominal surgery. The study adheres to the PRISMA statement for network meta-analysis and has been registered with PROSPERO. Moreover, the quality of evidence for each intervention regarding the primary outcomes was meticulously assessed using the GRADE criteria. Additionally, the inherent design of the network meta-analysis allows for the quantification of the effectiveness of each intervention for every outcome using the surface under the cumulative ranking (SUCRA).

## 5. Conclusions

Administration of probiotics in patients who have undergone abdominal surgery reduces the prevalence of post-operative ileus, the time to first flatus, the time to first defecation, and the prevalence of post-operative abdominal distension. Administration of synbiotics reduces the time to first flatus and the number of post-operative hospitalization days. In terms of treatment’s ranking, none of the interventions were universally superior to the others.

## Figures and Tables

**Table 1:** Direct and indirect effect estimates between the different interventions.

Time to first flatus			
**Probiotics**			−0.47 [−0.78 to −0.17]
0.05 [−0.48 to 0.59]	**Synbiotics**		−0.53 [−0.96 to −0.09]
−0.47 [−0.78 to −0.17]	−0.53 [−0.96 to −0.09]		**Placebo**
Time to first defecation			
**Probiotics**			−0.70 [−1.23 to −0.18]
−0.55 [−1.69 to 0.60]	**Synbiotics**		−0.15 [−1.17 to 0.87]
−0.70 [−1.23 to −0.18]	−0.15 [−1.17 to 0.87]		**Placebo**
Post-operative hospitalization days			
**Probiotics**			−0.76 [−2.57 to 1.04]
2.31 [−0.19 to 4.80]	**Synbiotics**		−3.07 [−4.80 to −1.34]
−0.84 [−5.27 to 3.59]	1.47 [−2.20 to 5.13]	**Prebiotics**	−1.60 [−5.66 to 2.45]
−0.76 [−2.57 to 1.04]	−3.07 [−4.80 to −1.34]	−1.60 [−5.66 to 2.45]	**Placebo**
Post-operative ileus			
**Probiotics**			0.38 [0.14–0.98]
0.47 [0.12–1.79]	**Synbiotics**		0.81 [0.31–2.08]
0.38 [0.14–0.98]	0.81 [0.31–2.08]		**Placebo**
Post-operative abdominal distension			
**Probiotics**			0.63 [0.48–0.82]
1.88 [0.39–8.98]	**Synbiotics**	1.55 [0.26–8.50]	0.33 [0.07–1.55]
0.80 [0.08–8.22]	1.55 [0.26–8.50]	**Prebiotics**	0.50 [0.05–5.08]
0.63 [0.48–0.82]	0.33 [0.07–1.55]	0.50 [0.05–5.08]	**Placebo**

**Table 2:** Surface under the cumulative ranking for each intervention, for the included outcomes.

Rank	SUCRA Time to First Flatus	SUCRA Time to First Defecation	SUCRA Post-Operative Hospitalization Days	SUCRA Post-Operative Ileus	SUCRA Post-Operative Abdominal Distension
1	Synbiotics (78.3)	Probiotics (91.1)	Synbiotics (91.6)	Probiotics (92.1)	Synbiotics (79.4)
2	Probiotics (71.2)	Synbiotics (39.5)	Prebiotics (54.7)	Synbiotics (40.2)	Probiotics (54.6)
3	Placebo (0.5)	Placebo (19.4)	Probiotics (39.6)	Ins (17.6)	Prebiotics (54.0)
4			Placebo (14.0)		Placebo (12.0)

## Data Availability

Not applicable.

## Supplementary Materials

The following supporting information can be downloaded at: https://www.mdpi.com/article/10.3390/jcm12124150/s1, Figure S1: Comparison-adjusted funnel plot; Table S1: Electronic search strategy; Table S2: Studies excluded at full-text review; Table S3: Characteristics of eligible studies; Table S4: Risk of bias; Table S5: GRADE evaluation.
