# Supplementary material for: The Efficacy of Probiotics, Prebiotics, and Synbiotics in Patients Who Have Undergone Abdominal Operation, in Terms of Bowel Function Post-Operatively: A Network Meta-Analysis
Source: J Clin Med. 2023 Jun 20;12(12):4150. doi: 10.3390/jcm12124150 (PMC10299319; doi:10.3390/jcm12124150)
Supplement: Supplementary file 1 [file jcm-12-04150-s001.zip › Table S1-search.pdf]

Supplementary Table S1. Electronic search strategy

Database	Search term (last search 17.3.2023)	Number
PubMed (All fields)	#1: synbiotics OR prebiotic OR probiotics OR probiotic OR prebiotics OR synbiotic	#1: 50244
	#2: operation OR surgery	#2: 6171562
	#3: gastrointestinal OR abdominal OR colorectal OR pancreatic OR laparotomy	#3: 1519369
	#4: #1 AND #2 AND #3	#4: 1506
Embase (All fields)	#1: synbiotics OR prebiotic OR probiotics OR probiotic OR prebiotics OR synbiotic	#1: 70802
	#2: operation OR surgery	#2: 8095628
	#3: gastrointestinal OR abdominal OR colorectal OR pancreatic OR laparotomy	#3: 2167303
	#4: #1 AND #2 AND #3	#4: 3102
Cochrane Library Trials (All fields)	#1: synbiotics OR prebiotic OR probiotics OR probiotic OR prebiotics OR synbiotic	#1: 10692
	#2: operation OR surgery	#2: 337993
	#3: gastrointestinal OR abdominal OR colorectal OR pancreatic OR laparotomy	#3: 131604
	#4: #1 AND #2 AND #3	#4: 448
Scopus (All fields)	#1: synbiotics OR prebiotic OR probiotics OR probiotic OR prebiotics OR synbiotic	#1: 86976
	#2: operation OR surgery	#2: 5073148
	#3: gastrointestinal OR abdominal OR colorectal OR pancreatic OR laparotomy	#3: 1812915
	#4: #1 AND #2 AND #3	#4: 1064
