# Supplementary material for: The Efficacy of Probiotics, Prebiotics, and Synbiotics in Patients Who Have Undergone Abdominal Operation, in Terms of Bowel Function Post-Operatively: A Network Meta-Analysis
Source: J Clin Med. 2023 Jun 20;12(12):4150. doi: 10.3390/jcm12124150 (PMC10299319; doi:10.3390/jcm12124150)
Supplement: Supplementary file 1 [file jcm-12-04150-s001.zip › Table S2- excluded.pdf]

Supplementary Table S2: Studies excluded at full-text review

Reference	Reason
1. Amitay EL, Carr PR, Gies A, Laetsch DC, Brenner H. Probiotic/Synbiotic Treatment and Postoperative Complications in Colorectal Cancer Patients: Systematic Review and Meta-analysis of Randomized Controlled Trials. Clin Transl Gastroenterol. 2020 Dec;11(12):e00268.	Meta-analysis
2. Zaharuddin L, Mokhtar NM, Muhammad Nawawi KN, Raja Ali RA. A randomized double-blind placebo-controlled trial of probiotics in post-surgical colorectal cancer. BMC Gastroenterol. 2019 Jul 24;19(1):131	No outcome of interest
3. Woodard GA, Encarnacion B, Downey JR, Peraza J, Chong K, Hernandez-Boussard T, Morton JM. Probiotics improve outcomes after Roux-en-Y gastric bypass surgery: a prospective randomized trial. J Gastrointest Surg. 2009 Jul;13(7):1198-204.	No outcome of interest
4. Wagner NRF, Ramos MRZ, de Oliveira Carlos L, da Cruz MRR, Taconeli CA, Filho AJB, Nassif LS, Schieferdecker MEM, Campos ACL. Effects of Probiotics Supplementation on Gastrointestinal Symptoms and SIBO after Roux-en-Y Gastric Bypass: a Prospective, Randomized, Double-Blind, Placebo-Controlled Trial. Obes Surg. 2021 Jan;31(1):143-150	No outcome of interest
5. Tang G, Huang W, Tao J, Wei Z. Prophylactic effects of probiotics or synbiotics on postoperative ileus after gastrointestinal cancer surgery: A meta-analysis of randomized controlled trials. PLoS One. 2022 Mar 1;17(3):e0264759.	Meta-analysis
6. Stephens JH, Hewett PJ. Clinical trial assessing VSL#3 for the treatment of anterior resection syndrome. ANZ J Surg. 2012 Jun;82(6):420-7.	No outcome of interest
7. Sadahiro S, Suzuki T, Tanaka A, Okada K, Kamata H, Ozaki T, Koga Y. Comparison between oral antibiotics and probiotics as bowel preparation for elective colon cancer surgery to prevent infection: prospective randomized trial. Surgery. 2014 Mar;155(3):493-503.	No outcome of interest
8. Zeng J, Ji Y, Liang B, Zhang G, Chen D, Zhu M, Wu S, Kuang W. The effect of pro/synbiotics on postoperative infections in colorectal cancer patients: A systematic review and meta-analysis. Complement Ther Clin Pract. 2021 May;43:101370	Meta-analysis
9. Mego M, Chovanec J, Vochyanova-Andrezalova I, Konkolovsky P, Mikulova M, Reckova M, Miskovska V, Bystricky B, Beniak J, Medvecova L, Lagin A, Svetlovska D, Spanik S, Zajac V, Mardiak J, Drgona L. Prevention of irinotecan induced diarrhea by probiotics: A randomized double blind, placebo controlled pilot study. Complement Ther Med. 2015 Jun;23(3):356-62.	No outcome of interest
10. McNaught CE, Woodcock NP, MacFie J, Mitchell CJ. A prospective randomised study of the probiotic Lactobacillus plantarum 299V on indices of gut barrier function in elective surgical patients. Gut. 2002 Dec;51(6):827-31	No outcome of interest

11. Xu L, Song M, Jiang Y, Li X. Comparative effectiveness of oral antibiotics, probiotics, prebiotics, and synbiotics in the prevention of postoperative infections in patients undergoing colorectal surgery: A network meta-analysis. Int Wound J. 2023 Feb;20(2):567-578. doi: 10.1111/iwj.13888	Meta-analysis
12. Liu ZH, Huang MJ, Zhang XW, Wang L, Huang NQ, Peng H, Lan P, Peng JS, Yang Z, Xia Y, Liu WJ, Yang J, Qin HL, Wang JP. The effects of perioperative probiotic treatment on serum zonulin concentration and subsequent postoperative infectious complications after colorectal cancer surgery: a double-center and double-blind randomized clinical trial. Am J Clin Nutr. 2013 Jan;97(1):117-26	No outcome of interest
13. Lee JY, Chu SH, Jeon JY, Lee MK, Park JH, Lee DC, Lee JW, Kim NK. Effects of 12 weeks of probiotic supplementation on quality of life in colorectal cancer survivors: a double-blind, randomized, placebo-controlled trial. Dig Liver Dis. 2014 Dec;46(12):1126-32	No outcome of interest
14. Wu XD, Xu W, Liu MM, Hu KJ, Sun YY, Yang XF, Zhu GQ, Wang ZW, Huang W. Efficacy of prophylactic probiotics in combination with antibiotics versus antibiotics alone for colorectal surgery: A meta-analysis of randomized controlled trials. J Surg Oncol. 2018 Jun;117(7):1394-1404.	Meta-analysis
15. Grąt M, Wronka KM, Lewandowski Z, Grąt K, Krasnodębski M, Stypułkowski J, Hołówko W, Masior Ł, Kosińska I, Wasilewicz M, Raszeja-Wyszomirska J, Rejowski S, Bik E, Patkowski W, Krawczyk M. Effects of continuous use of probiotics before liver transplantation: A randomized, double-blind, placebo-controlled trial. Clin Nutr. 2017 Dec;36(6):1530-1539	No outcome of interest
16. Dikeocha IJ, Al-Kabsi AM, Eid EEM, Hussin S, Alshawsh MA. Probiotics supplementation in patients with colorectal cancer: a systematic review of randomized controlled trials. Nutr Rev. 2021 Dec 8;80(1):22-49	Systematic Review
17. Chowdhury AH, Adiamah A, Kushairi A, Varadhan KK, Krznaric Z, Kulkarni AD, Neal KR, Lobo DN. Perioperative Probiotics or Synbiotics in Adults Undergoing Elective Abdominal Surgery: A Systematic Review and Meta-analysis of Randomized Controlled Trials. Ann Surg. 2020 Jun;271(6):1036-1047	Meta-analysis
18. Golkhalkhali B, Rajandram R, Paliany AS, Ho GF, Wan Ishak WZ, Johari CS, Chin KF. Strain-specific probiotic (microbial cell preparation) and omega-3 fatty acid in modulating quality of life and inflammatory markers in colorectal cancer patients: a randomized controlled trial. Asia Pac J Clin Oncol. 2018 Jun;14(3):179-191	No outcome of interest
19. Chen Y, Qi A, Teng D, Li S, Yan Y, Hu S, Du X. Probiotics and synbiotics for preventing postoperative infectious complications in colorectal cancer patients: a systematic review and meta-analysis. Tech Coloproctol. 2022 Jun;26(6):425-436	Meta-analysis
20. Kakaei F, Shahrasbi M, Kermani TA, et al. Assessment of probiotic effects on colo- rectal surgery complications: a double blinded, randomized clinical trial. Biomed Res Ther. 2019;6:3067–3072.	No outcome of interest
21. Gianotti L, Morelli L, Galbiati F, Rocchetti S, Coppola S, Beneduce A, Gilardini C, Zonenschain D, Nespoli A, Braga M. A randomized double-blind trial on perioperative administration of probiotics in colorectal cancer patients. World J Gastroenterol. 2010 Jan 14;16(2):167-75.	No outcome of interest
22. Kahn J, Pregartner G, Schemmer P. Effects of both Pro- and Synbiotics in Liver Surgery and Transplantation with Special Focus on the Gut-Liver Axis-A Systematic Review and Meta-Analysis. Nutrients. 2020 Aug 15;12(8):2461.	Meta-analysis

23. Calikoglu F, Barbaros U, Uzum AK, Tutuncu Y, Satman I. The Metabolic Effects of Pre-probiotic Supplementation After Roux-en-Y Gastric Bypass (RYGB) Surgery: a Prospective, Randomized Controlled Study. Obes Surg. 2021 Jan;31(1):215-223	No outcome of interest
24. Zhang Y, Chen J, Wu J, Chalson H, Merigan L, Mitchell A. Probiotic use in preventing postoperative infection in liver transplant patients. Hepatobiliary Surg Nutr. 2013 Jun;2(3):142-7.	Not RCT
25. Reddy BS, Macfie J, Gatt M, Larsen CN, Jensen SS, Leser TD. Randomized clinical trial of effect of synbiotics, neomycin and mechanical bowel preparation on intestinal barrier function in patients undergoing colectomy. Br J Surg. 2007 May;94(5):546-54	No outcome of interest
26. Arumugam S, Lau CS, Chamberlain RS. Probiotics and Synbiotics Decrease Postoperative Sepsis in Elective Gastrointestinal Surgical Patients: a Meta-Analysis. J Gastrointest Surg. 2016 Jun;20(6):1123-31	Meta-analysis
27. Pitsouni E, Alexiou V, Saridakis V, Peppas G, Falagas ME. Does the use of probiotics/synbiotics prevent postoperative infections in patients undergoing abdominal surgery? A meta-analysis of randomized controlled trials. Eur J Clin Pharmacol. 2009 Jun;65(6):561-70.	Meta-analysis
28. Okazaki M, Matsukuma S, Suto R, Miyazaki K, Hidaka M, Matsuo M, Noshima S, Zempo N, Asahara T, Nomoto K. Perioperative synbiotic therapy in elderly patients undergoing gastroenterological surgery: a prospective, randomized control trial. Nutrition. 2013 Oct;29(10):1224-30	No outcome of interest
29. Lytvyn L, Quach K, Banfield L, Johnston BC, Mertz D. Probiotics and synbiotics for the prevention of postoperative infections following abdominal surgery: a systematic review and meta-analysis of randomized controlled trials. J Hosp Infect. 2016 Feb;92(2):130-9	Meta-analysis
30. Tang G, Zhang L, Huang W, Wei Z. Probiotics or Synbiotics for Preventing Postoperative Infection in Hepatopancreatobiliary Cancer Patients: A Meta-Analysis of Randomized Controlled Trials. Nutr Cancer. 2022;74(10):3468-3478	Meta-analysis
31. Rodríguez-Padilla Á, Morales-Martín G, Pérez-Quintero R, Gómez-Salgado J, Balongo-García R, Ruiz-Frutos C. Postoperative Ileus after Stimulation with Probiotics before Ileostomy Closure. Nutrients. 2021 Feb 15;13(2):626	Intervention method not of interest
32. Eguchi S, Takatsuki M, Hidaka M, Soyama A, Ichikawa T, Kanematsu T. Perioperative synbiotic treatment to prevent infectious complications in patients after elective living donor liver transplantation: a prospective randomized study. Am J Surg. 2011 Apr;201(4):498-502	No outcome of interest
33. Tang G, Zhang L, Tao J, Wei Z. Effects of Perioperative Probiotics and Synbiotics on Pancreaticoduodenectomy Patients: A Meta-Analysis of Randomized Controlled Trials. Front Nutr. 2021 Aug 13;8:715788	Meta-analysis
34. Anderson AD, McNaught CE, Jain PK, MacFie J. Randomised clinical trial of synbiotic therapy in elective surgical patients. Gut. 2004 Feb;53(2):241-5	No outcome of interest
35. Veziant J, Bonnet M, Occean BV, Dziri C, Pereira B, Slim K. Probiotics/Synbiotics to Reduce Infectious Complications after Colorectal Surgery: A Systematic Review and Meta-Analysis of Randomised Controlled Trials. Nutrients. 2022 Jul 26;14(15):3066.	Meta-analysis

36. Iida H, Sasaki M, Maehira H, Mori H, Yasukawa D, Takebayashi K, Kurihara M, Bamba S, Tani M. The effect of preoperative synbiotic treatment to prevent surgical-site infection in hepatic resection. J Clin Biochem Nutr. 2020 Jan;66(1):67-73	Synbiotics both in control
37. He D, Wang HY, Feng JY, Zhang MM, Zhou Y, Wu XT. Use of pro-/synbiotics as prophylaxis in patients undergoing colorectal resection for cancer: a meta-analysis of randomized controlled trials. Clin Res Hepatol Gastroenterol. 2013 Sep;37(4):406-15.	Meta-analysis
38. Rayes N, Pilarski T, Stockmann M, Bengmark S, Neuhaus P, Seehofer D. Effect of pre- and probiotics on liver regeneration after resection: a randomised, double-blind pilot study. Benef Microbes. 2012 Sep;3(3):237-44	No outcome of interest
39. Theodoropoulos GE, Memos NA, Peitsidou K, Karantanos T, Spyropoulos BG, Zografos G. Synbiotics and gastrointestinal function-related quality of life after elective colorectal cancer resection. Ann Gastroenterol. 2016 Jan-Mar;29(1):56-62	No outcome of interest
