# Supplementary material for: The Efficacy of Probiotics, Prebiotics, and Synbiotics in Patients Who Have Undergone Abdominal Operation, in Terms of Bowel Function Post-Operatively: A Network Meta-Analysis
Source: J Clin Med. 2023 Jun 20;12(12):4150. doi: 10.3390/jcm12124150 (PMC10299319; doi:10.3390/jcm12124150)
Supplement: Supplementary file 1 [file jcm-12-04150-s001.zip › Table S3.pdf]

Supplementary Table S3: Characteristics of eligible studies

Probiotics

Study	Type of study	Sample	Age	Male	Primary Disease/Condition	Type of surgery	Intervention group	Control group	Treated days (pre + post-surgery)	Outcome
Liu, 2010	DB, RCT	I: 50<br>C: 50	69	66	Colorectal cancer	Radical colorectomy	Encapsulated bacteria containing Lactobacillus plantarum (CGMCC No. 1258, cell count 10^{11} CFU/g), Lactobacillus acidophilus (LA-11, cell count 7.0×10^{10} CFU/g) and Bifidobacterium longum (BL-88, cell count 5.0×10^{10} CFU/g), 2 g/day	Placebo	6 days preoperative + postoperative day 1–10	TD, AD, LOP
Diepenhorst, 2010	RCT	I: 10<br>C: 10	64	10	Periampullary or ampullary pancreatic cancer	PD	Ecologic641 consisting of six probiotic strains (3g twice a day)	No intervention	7 days preoperative + postoperative 1-7	PI
Zhang, 2012	DB, RCT	I: 30<br>C: 30	65	24	Colorectal cancer	Radical colorectomy	Bifid triple viable capsules, each of which contained 0.21 g (10^8 CFU/g) of B longum, L acidophilus and Enterococcus faecalis (one capsule 3 times a day).	Placebo	3-5 days preoperative	PI
Mangell, 2012	DB, RCT	I: 32<br>C: 32	72	36	Colorectal cancer	Colon resection	Daily consumption of 10^{11} CFU of Lp 299v	Placebo	8 days preoperative + postoperative 1-5	TD, TF
Chen, 2014	DB, RCT	I: 35<br>C: 35	N	N	Colorectal cancer	Radical colorectomy	Probiotics preparation consisting of two combined live bacteria	Placebo	5 days preoperative + postoperative day 1–7	TD, TF, AD
Liu, 2015	DB, RCT	I: 66<br>C: 68	63	70	Colorectal liver metastases	Colorectal liver metastases surgery	Encapsulated admixture of three probiotics bacteria, composed of LP (CGMCC No.1258, cell count $\geq 10^{11}$ CFU/g), LA-11 (cell count $\geq 7.0 \times 10^{10}$ CFU/g) and BL-88 (cell count $\geq 5.0 \times 10^{10}$ CFU/g) (2 g a day)	Placebo	6 days preoperative + postoperative day 1–10	AD, LOP, TD
Mizuta, 2015	RCT	I: 31<br>C: 29	70	35	Colorectal cancer	Colorectal cancer resection	A sachet containing 2 g of B. longum BB536 powder (approximately 5×10^{10} colony-forming units/2 g) daily	SC	7-14 days preoperative + postoperative day 1–14	LOP
Kotzampassi 2015	DB, RCT	I: 84<br>C: 80	66	115	Colorectal cancer	Colon resection	Lactobacillus acidophilus LA-5 1.75 9 10 9 CFU, Lactobacillus plantarum 0.5×10^9 CFU, Bifidobacterium lactis BB-12 1.75×10^9 CFU and Saccharomyces boulardii 1.5×10^9 CFU per capsule	Placebo	postoperative day 1–14	LOP, TD

Consoli, 2015	RCT	I: 15<br>C: 18	55	15	Colorectal cancer, other diseases	Elective colon resection	Once-daily oral lyophilized yeast capsule with 100 mg (0.5 × 10^9 CGU/g) of S boulardii (Merck S.A., Biocodex, France).	No intervention	1-7 days preoperative	LOP
Yang, 2016	DB, RCT	I: 30<br>C: 30	63	27	Colorectal Cancer	Colorectal cancer surgery	Combined probiotics containing Bifidobacterium longum (≥ 1.0×10^7 CFU/g), Lactobacillus acidophilus (≥ 1.0 ×10^7 CFU/g), and Enterococcus faecalis (≥ 1.0 ×107 CFU/g) (2g, 3 times a day)	Placebo	5 days preoperative + postoperative day 1–7	TF, TD, LOP, AB
Tan, 2016	DB, RCT	I: 20<br>C: 20	66	24	Colorectal Cancer	Colorectal cancer surgery	An orange-flavored granular powder, containing 30 billion colony-forming units of highly compatible, acid- and bile-resistant strains of Lactobacillus acidophilus, Lactobacillus casei, Lactobacillus lactis, Bifidobacterium bifidum, Bifidobacterium longum, and Bifidobacterium infantis (twice daily)	Placebo	7 days preoperative	LOP
Xie, 2018	RCT	I: 70<br>C: 70	68	67	Gastric cancer	Distal gastrectomy	Probiotics three times a day	SC	Postoperative day 1–8	TF, LOP
Xu, 2018	RCT	I: 30<br>C: 30	62	38	Colorectal cancer	Colorectal cancer surgery	Bifidus-triple viable preparation daily	SC	Postoperative day 1–7	TF
Bajramagic, 2019	RCT	I: 39<br>C: 39	N	N	Colorectal cancer	Colorectal cancer surgery	Probiotic capsules contain eight bacterial cultures (Lactobacillus acidophilus, Lactobacillus casei, Lactobacillus plantarum, Lactobacillus rhamnosus, Bifidobacterium lactis, Bifidobacterium bifidum, Bifidobacterium breve, Streptococcusthermophilus) (one capsule twice daily)	SC	Postoperative day 3–30	PI
Park, 2020	DB, RCT	I: 29<br>C: 30	61	32	Colorectal cancer	Colorectal cancer surgery	Two g probiotic powder contained three probiotic strains (twice daily)	Placebo	7 days preoperative + postoperative day 1–21	PI
Zeng, 2020	RCT	I: 54<br>C: 53	56	71	Gastric cancer	Gastrectomy	Bifid triple viable (0.42-0.84g twice daily)	SC	Postoperative day 1–7	TF, TD
Yoon, 2020	DB, RCT	I: 19<br>C: 17	60	23	Rectal cancer	Ileostomy reversal	Three-week course of probiotics at a dose of 1×10^{10} lactic acid bacteria (Lactobacillus plantarum) one sachet per day orally. Only one day before the operation, the patients received two sachets.	Placebo	21 days preoperative+ 21 days postoperative	TF, TD
Fowarski, 2021	RCT	I: 20<br>C: 20	N	25	Pancreatic cancer/ Chronic Pancreatitis	Pylorus-preserving Longmire-Traverso PD with pancreatojejunostomy	L. rhamnosus GG- one capsule containing 6 million CFU of this PB strain every 12 hours from the day of the surgery for 30 days	SC	Day of surgery + postoperative day 1–30	TF, TD, LOP
Wang, 2021	DB, RCT	I: 26<br>C: 25	70	N	Colorectal Cancer	Colorectal cancer surgery	Probiotic capsules (0.84 g) twice daily from admission until discharge. The probiotic capsule (BIFICO, Sine Pharmaceuticals, Shanghai, China) contained Bi fi dobacterium longum (≥ 1.0 × 10^7 CFU/capsule), Lactobacillus acidophilus (≥ 1.0 × 10^7 CFU/capsule), and Enterococcus faecalis (≥ 1.0 × 10^7 CFU/capsule).	Placebo	3-5 days preoperative + Postoperative day 1–7	TF, TD, LOP

Synbiotics

Study	Type of study	Sample	Age	Male	Primary Disease	Type of surgery	Intervention group	Control group	Treated days (pre + post-surgery)	Outcome
Rayes, 2002	RCT	I: 30<br>C: 30	61	30	Abdominal disease	Major abdominal surgery	Lactobacillus plantarum 299 in a dose of 10^9 and oat fiber twice daily through the feeding tube during the first 4 days.	Placebo	Postoperative day 1–5	LOP
Rayes, 2002	RCT	I: 31<br>C: 32	50	30	Liver disease	Liver transplantation	Lactobacillu plantarum 299 (AB Probi, Lund, Sweden) in a dose of 10^9 and oat fiber were added twice daily via the feeding tube.	Placebo	Postoperative day 1–12	LOP
Rayes, 2005	DB, RCT	I: 33<br>C: 33	51	38	Cirrhosis	Liver transplantation	Synbiotic composition of pre- and probiotics was administered twice daily via the feeding tube or orally. Each dose of the combination contains four different LAB: 10 10 Pediacoccus pentosaceus 5-33:3 (dep. no. LMG P-20608), Leuconostoc mesenteroides 77:1 (dep. no. LMG P-20607), Lactobacillus paracasei ssp. paracasei F19 (dep. no. LMG P-17806) and L. plantarum 2362 (dep. no. LMG P-20606) plus four bioactive fibers: 2.5 g of each betaglucan, inulin, pectin and resistant starch, totally 10 g/dose, or 20 g/day.	PRE (Four bioactive fibers))	Postoperative day 1–14	LOP
Kanazawa, 2005	RCT	I: 21<br>C: 23	64	29	Biliary cancer	Combined liver and extrahepatic bile duct resection with hepaticojejunostomy	Yakult BL Seichōyaku (3 g/day) and GOS (12 g/day). Yakult BL Seichōyaku contains: 1×10^8 living Bifidobacterium breve strain Yakult and 1×10^8 living Lactobacillus casei strain Shirota/g	SC	Postoperative day 1–14	LOP
Rayes, 2007	DB, RCT	I: 40<br>C: 40	58	45	Pancreatic cancer/ Chronic Pancreatitis	Pylorus-preserving PD	Synbiotic composition of pre- and probiotics was administered twice daily via the feeding tube or orally. Each dose of the combination contains 4 different lactic acid bacteria: 10^{10} Pediacoccus pentosaceus 5–33:3 (dep.nr LMG P-20608), Leuconostoc mesenteroides 77:1 (dep.nr LMG P-20607), Lactobacillus paracasei subspecies paracasei F19 (dep.nr LMG P-17806), and Lactobacillus plantarum 2362 (dep.nr LMG P-20606) plus 4 bioactive fibers: 2.5 g of each betaglucan, inulin, pectin, and resistant starch, totally 10 g per dose, or 20 g per day	PRE (Four bioactive fibers)	1 day preoperative + postoperative day1-8	LOP,AD

Usami, 2010	RCT	I: 32<br>C: 29	66	55	Primary or metastatic liver cancer	Liver resection	Yakult BL Seichōyaku (3 g/day) and GOS (10g/day). Yakult BL Seichōyaku contains: 1×10^8 living Bifidobacterium breve strain Yakult and 1×10^8 living Lactobacillus casei strain Shirota/g.	SC	14 days preoperative + postoperative day 3–14	LOP
Horvat, 2010	DB, RCT	I: 20<br>PRE: 28<br>C: 20	63	30	Colorectal cancer	Elective colorectal	Combination of pro- and prebiotics was the multi-strain/ multi-fiber Synbiotic 2000™. four lactobacilli, one from each of four main genera: 10^{10} of Pediacoccus pentosaceus 5-33:3, 10^{10} of Leuconostoc mesenteroides 32–77:1, 10^{10} of Lactobacillus paracasei subsp. paracasei 19 and 10^{10} of Lactobacillus plantarum 2362. Each dose thus contains a total of 40 billion lactobacilli plus 10 g of well-studied bioactive plant fibers (2.5 g betaglucan, 2.5 g inulin, 2.5 g pectin, 2.5 g resistant starch) packed in sachets that are mixed with water.	Placebo & PRE	1-3 day preoperative	TF, LOP
Tanaka, 2012	RCT	I: 30<br>C: 34	62	51	Esophageal cancer	Esophagectomy	Yakult BL Seichōyaku (3 g/day) and GOS (15g/day). Yakult BL Seichōyaku contains: 1×10^8 living Bifidobacterium breve strain Yakult and 1×10^8 living Lactobacillus casei strain Shirota/g.	SC	Postoperative day 1–21	TD, TF
Russolillo, 2014	RCT	I: 20<br>C: 20	63	24	Hepato-biliary pancreatic cancer	Hepato-biliary pancreatic surgery	Prebiotic (Mediabase srl., Prato, Italy) in a dose of one sachet twice a day for at least 1 week preoperatively. Postoperatively, the medication was reintroduced as tolerated and continued until discharge from hospital. One 4.5 gr sachet of Prebiotic contains at least 10^{10} living Bifidobacterium bifidum, 10^{10} living Streptococcus thermophilus, 10^{10} living Streptococcus salivarius, 3 x 10^9 Lactobacillus acidophilus, 10^{10} living Lactobacillus casei, 10^{10} living Lactobacillus bulgaricus and galacto-oligosaccharides (4.5g).	SC	7 day preoperative + until discharge	LOP
Krebs, 2015	DB, RCT	I: 20<br>C: 16	66	22	Colorectal cancer	Colorectal surgery	One sachet consists 10^{11} of each of four LAB: Pediacoccus pentosaceus 5–33:3, Leuconostoc mesenteroides 32–77:1, Lactobacillus paracasei subsp paracasei 19, and Lactobacillus plantarum 2362. This makes 400 billion LAB per dose. Also included in the sachet is 2.5 g of each of the four fermentable fibres (one sachet twice a day)	Placebo	1-3 days preoperative	TD, TF, DS
Yokoyama, 2014	RCT	I: 21<br>C: 21	66	37	Oesophageal cancer	Oesophagectomy	One 80-ml bottle of Yakult 400, which contained at least 4×10^{10} living Lactobacillus casei strain Shirota; one 100-ml bottle of MILMIL-S, which contained at least 1×10^{10} living Bifidobacterium breve strain Yakult; and 15 g GOS (Daily)	SC	7 days preoperative + postoperative day 1–14	LOP

Komatsu, 2015	RCT	I: 168<br>C: 194	67	210	Colorectal cancer	Colorectal surgery	One 80-ml bottle of Yakult Ace, which contained at least 4 × 10^{10} living Lactobacillus casei strain Shirota with 2.5 g GOS; and one 100-ml bottle of MILMIL-S, which contained at least 1 × 10^{10} living Bifidobacterium breve strain Yakult (Daily)	SC	7-11 days preoperative + postoperative day 2–7	PI
Sommacal, 2015	DB, RCT	I: 23<br>C: 23	60	N	Periampullary cancer	PD	Lactobacillus acidophilus 10, 1 × 10^9 CFU, Lactobacillus rhamnosus HS 111, 1 × 10^9 CFU, Lactobacillus casei 10, 1 × 10^9 CFU, Bifidobacterium bifidum, 1 × 10^9 CFU + fructooligosaccharides 100 mg (twice daily)	Placebo	4 days preoperative + postoperative day 1–10	LOP
Rammohan, 2015	RCT	I: 39<br>C: 36	43	48	Chronic pancreatitis	Frey procedure	Specific composition of prebiotics and probiotics (synbiotics) [Streptococcus faecalis T-110-60 million, Clostridium butyricum TOA-4 million, Bacillus mesentericus TO-A-2 million, Lactobacillus sporogenes-100 million, Fructooligosaccharides (Bifiliac HP, Tablets, India)] was administered thrice daily orally.	Placebo	5 days preoperative + postoperative day 1–10	LOP
Yokoyama, 2016	RCT	I: 22<br>C: 22	65	12	Biliary pancreatic cancer, Chronic pancreatitis	PD	80-ml bottle of Yakult 400 (Yakult Honsha, Tokyo, Japan), which contained 4 × 10^{10} living Lactobacillus casei strain Shirota; one 100-ml bottle of MILMIL S (Yakult Honsha), which contained 1 × 10^{10} living Bifidobacterium breve strain Yakult; and 15 g of galacto-oligosaccharides (Oligomate S-HP; Yakult Honsha)	SC	7 days preoperative + postoperative day 1–14	LOP
Flesch, 2017	DB, RCT	I: 49<br>C: 42	62	37	Colorectal cancer	Colorectal cancer surgery	Two sachets twice a day L bacillus acidophilus NCFM (10^9), Lactobacillus rhamnosus HN001 (10^9), Lactobacillus paracasei LPC-37 (10^9), Bifidobacterium lactis HN019 (10^9) and fructo -oligosaccharides (FOS) 6g.	Placebo	5 days preoperative + postoperative day 1–14	LOP
Zhao, 2017	RCT	I: 40<br>C: 40	65	38	Gastric cancer	Gastrectomy	Six g of live bifidobacterium and lactobacillus in tablets, 30 g of Shen Jia (daily).	SC	postoperative day 1–7	TF, AD, LOP
Polakowski, 2018	DB, RCT	I: 36<br>C: 37	60	39	Colorectal cancer	Colorectal cancer surgery	Simbioflora, a dietary supplement comprising 6g of fructooligosaccharide, and the probiotics Lactobacillus acidophilus NCFM, Lactobacillus rhamnosus HN001, Lactobacillus casei LPC-37 and Bifidobacterium lactis HN019 in the concentration of 10^9 (6g twice daily)	Placebo	1-7 days preoperative	LOP

CFU, colony forming units; C, Control group; DB, Double blind; I, Intervention group; GOS, galacto-oligosaccharides; PD, pancreatoduodenectomy N; not available; RCT, randomized controlled trial; SC, standard care; TF, time to first flatus; TD: time to first defecation; LOP: Length of postoperative hospital stay; PI: Postoperative ileus; AD; abdominal distension; Prebiotics; PRE.
