# Supplementary material for: The Efficacy of Probiotics, Prebiotics, and Synbiotics in Patients Who Have Undergone Abdominal Operation, in Terms of Bowel Function Post-Operatively: A Network Meta-Analysis
Source: J Clin Med. 2023 Jun 20;12(12):4150. doi: 10.3390/jcm12124150 (PMC10299319; doi:10.3390/jcm12124150)
Supplement: Supplementary file 1 [file jcm-12-04150-s001.zip › Table S4-RoB .pdf]

	D1	D2	D3	D4	D5	Overall
Rayes 2002	+	+	+	?	+	?
Rayes (Liver) 2002	+	+	X	?	+	X
Rayes 2005	?	+	+	+	+	?
Kanazawa 2005	X	?	+	+	+	X
Rayes 2007	+	+	+	+	+	+
Usami 2010	+	?	+	+	+	?
Liu 2010	+	+	+	+	+	+
Diepenhorst 2010	+	?	+	+	+	?
Horvat 2010	+	+	+	+	+	+
Tanaka 2012	X	?	+	+	+	X
Zhang 2012	X	?	+	+	+	X
Mangell 2012	+	+	X	+	+	X
Russolillo 2014	+	+	X	?	+	X
Chen 2014	X	+	+	+	+	X
Yokoyama 2014	X	?	+	+	+	X
Sommacal 2015	+	+	+	+	+	+
Komatsu 2014	+	?	+	+	+	?
Rammohan 2015	+	+	?	?	+	?
Kotzampassi 2015	+	+	+	+	?	?
Liu 2015	+	?	+	+	+	?
Mizuta 2015	X	?	+	+	+	X
Consoli 2015	+	?	+	+	+	?
Krebs 2015	+	?	+	+	+	?
Yang 2016	+	?	+	+	+	?
Tan 2016	+	+	+	+	+	+
Yokoyama 2016	+	X	+	+	+	X
Zhao 2017	X	?	+	+	+	X
Flesch 2017	+	+	?	+	+	?
Polakowski 2017	+	+	+	+	+	+
Xie 2018	X	?	+	+	+	X
Xu 2018	X	?	+	+	+	X
Bajramagic 2019	X	?	+	+	+	X
Park 2020	+	+	+	+	+	+
Zeng 2020	X	?	+	+	+	X
Yoon 2020	+	+	+	+	+	+
Fowarski 2021	+	X	+	+	+	X
Wang 2021	+	+	+	+	+	+

Supplementary Table S4. Risk of Bias of included studies. D1: Bias arising from the randomization process. D2: Bias due to deviations from intended intervention. D3: Bias due to missing outcome data. D4: Bias in measurement or the outcome. D5: Bias in selection of th[illegible]eported result. Hi[illegible] Son[illegible] concerns Low
