# Supplementary material for: The Efficacy of Probiotics, Prebiotics, and Synbiotics in Patients Who Have Undergone Abdominal Operation, in Terms of Bowel Function Post-Operatively: A Network Meta-Analysis
Source: J Clin Med. 2023 Jun 20;12(12):4150. doi: 10.3390/jcm12124150 (PMC10299319; doi:10.3390/jcm12124150)
Supplement: Supplementary file 1 [file jcm-12-04150-s001.zip › Table S5 GRADE.pdf]

Table S5a. GRADE evaluation for the primary outcome of post-operative ileus.

Comparison	№ of participants (studies)	Relative Risk (95% CI)	Certainty of the evidence (GRADE)	Comments
Probiotics vs Placebo (direct evidence)	217<br>(4 studies)	**0.38 [0.14, 0.98]**	Low	Within study bias<br>Heterogeneity
Probiotics vs Placebo (Network MA)		**0.38 [0.14, 0.98]**	Low	Within study bias<br>Heterogeneity
Synbiotics vs Placebo (direct evidence)	362<br>(1 study)	**0.81 [0.31, 2.08]**	Moderate	Imprecision
Synbiotics vs Placebo (Network MA)		**0.81 [0.31, 2.08]**	Moderate	Imprecision
Probiotics vs Synbiotics (Network MA)		**0.47 [0.12, 1.79]**	Moderate	Imprecision

Table S5b. GRADE evaluation for the primary outcome of time to first flatus.

Comparison	№ of participants (studies)	Mean Difference (95% CI)	Certainty of the evidence (GRADE)	Comments
Probiotics vs Placebo (direct evidence)	521<br>(7 study)	**-0.47 [-0.78, -0.17]**	Moderate	Within study bias
Probiotics vs Placebo (Network MA)		**-0.47 [-0.78, -0.17]**	Moderate	Within study bias
Synbiotics vs Placebo (direct evidence)	180<br>(3 study)	**-0.53 [-0.96, -0.09]**	Low	Within study bias<br>Heterogeneity
Synbiotics vs Placebo (Network MA)		**-0.53 [-0.96, -0.09]**	Low	Within study bias<br>Heterogeneity
Probiotics vs Synbiotics (Network MA)		**-0.55 [-1.69, 0.60]**	Low	Within study bias<br>Imprecision
