# Supplementary figures and images for: The Efficacy of Probiotics, Prebiotics, and Synbiotics in Patients Who Have Undergone Abdominal Operation, in Terms of Bowel Function Post-Operatively: A Network Meta-Analysis
Source: J Clin Med. 2023 Jun 20;12(12):4150. doi: 10.3390/jcm12124150 (PMC10299319; doi:10.3390/jcm12124150)

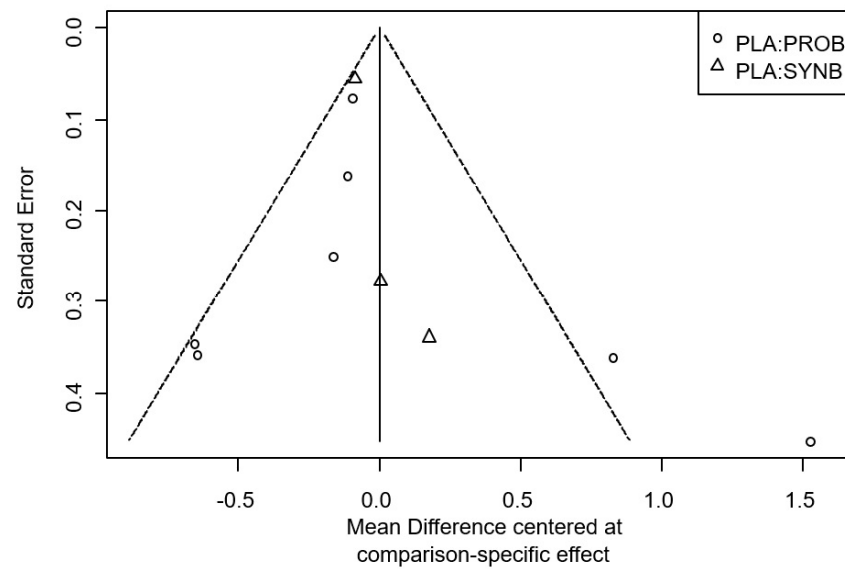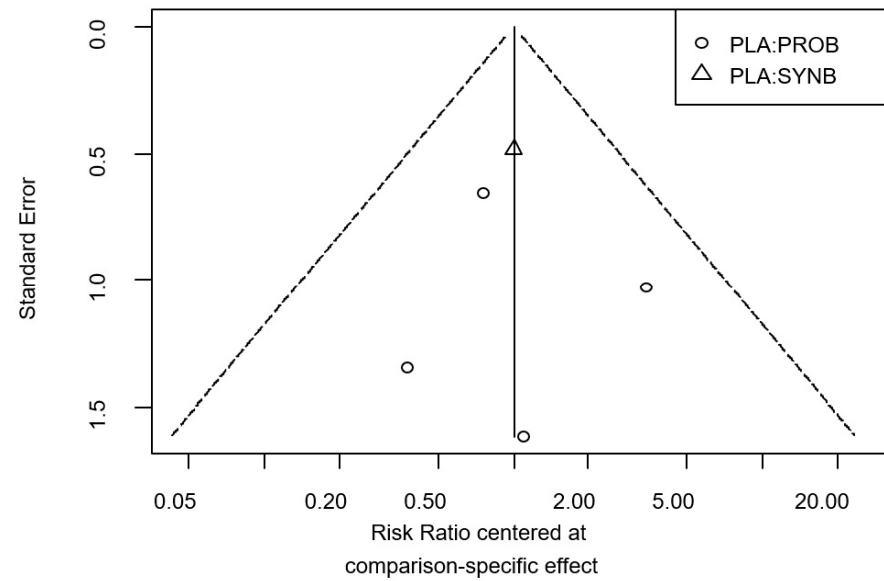

**Supplementary Figure S1. Comparison-adjusted funnel plot.**

Supplement: Supplementary file 1 [file jcm-12-04150-s001.zip › Figure S1.pdf]
